# Are Ar_3_SbCl_2_ Species Lewis Acidic? Exploration of the Concept and Pnictogen Bond Catalysis Using a Geometrically Constrained Example

**DOI:** 10.1021/acs.organomet.2c00565

**Published:** 2023-01-30

**Authors:** Jesse E. Smith, François P. Gabbaï

**Affiliations:** Department of Chemistry, Texas A&M University, College Station, Texas 77843, United States

## Abstract

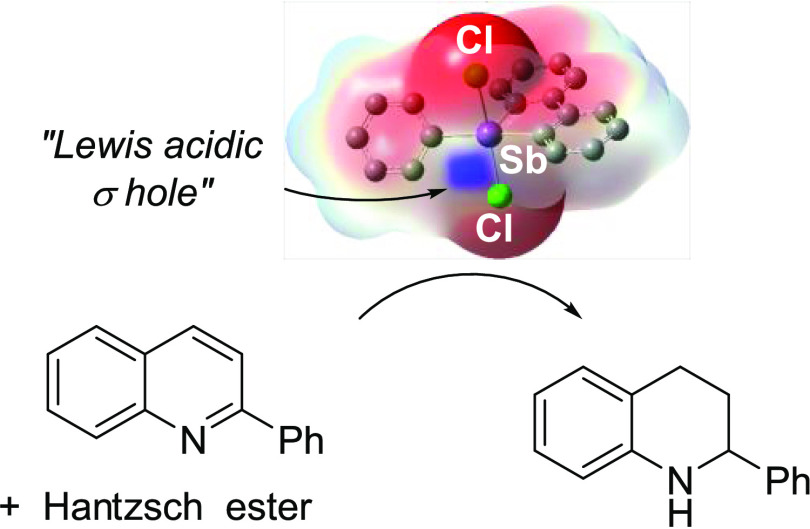

As part of our investigations into the Lewis acidic behavior of antimony derivatives, we have decided to study the properties of 5-phenyl-5,5-dichloro-λ^5^-dibenzostibole (**1**), a dichlorostiborane with an antimony atom confined to a five-membered heterocycle. Our work shows that the resulting geometrical constraints elevate the Lewis acidity of the antimony atom, as confirmed by the crystal structure of **1**-THF and the solution study of the interaction of **1** with Ph_3_PO. The enhanced Lewis acidic properties of **1**, which exceed those of simple dichlorostiboranes such as Ph_3_SbCl_2_, also become manifest in pnictogen bonding catalysis experiments involving the reductions of imines with Hantzsch ester. The influence of geometrical constraints in the chemistry of this compound is also supported by a computational activation strain analysis as well as by an energy decomposition analysis of a model Me_3_PO adduct.

## Introduction

**Chart 1:**
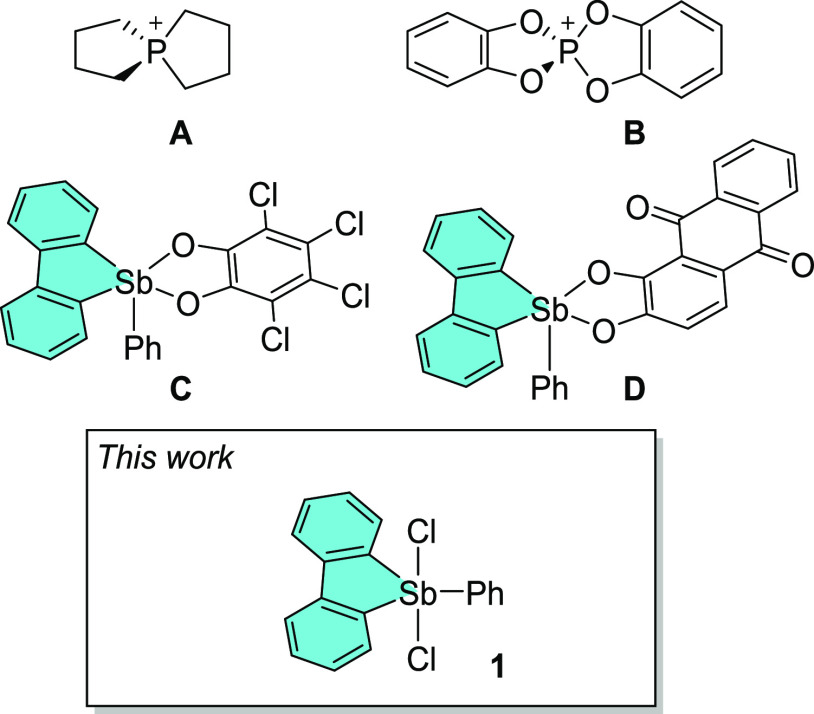
Examples of Lewis Acidic Bicyclic Phosphonium Cations (**A** and **B**) and λ^5^-Dibenzostibole (**C** and **D**), along with the Structure of 5-Phenyl-5,5-dichloro-λ^5^-dibenzostibole (**1**)

Geometrical constraints can be used to manipulate the electronic structure of main group derivatives and thus fine-tune their reactivity. In the context of Lewis acid chemistry, it has long been known that simply incorporating silicon into a rigid five-membered structure elevates its Lewis acidity.^1^ Similar strategies have been employed in the chemistry of group 13 compounds as in the case of distorted or pyramidalyzed boranes, with externally exposed “vacant” oribtals.^2^ The same concepts have driven a surge of efforts in pnictogen chemistry, where ligand-imposed geometrical constraints have been used to adjust not only the Lewis acidity of the main group element but also its redox reactivity.^3^ Examples of such compounds include bicyclic phosphonium cations such as **A**(4) and **B**,^5^ which display remarkable, group 15-centered Lewis acidity (Chart 1).^6^ The properties of such derivatives originate from the constraints imposed by the cyclic structure. These constraints limit relaxation of the endocyclic angle, leading to a ground-state destabilization of the Lewis acid and thus providing a greater exothermic drive for the coordination of a Lewis base. The same ground-state destabilization argument explains the differing fluoride anion affinities (FIAs) computed by Morokuma and co-workers for PF_5_ at its ground-state *D*_3*h*_ geometry and distorted *C*_4*v*_ square pyramidal geometry. With the latter lying 4.3 kcal/mol over the former,^7^ the FIA of PF_5_ at the *C*_4*v*_ geometry (96.2 kcal/mol) exceeds that of the *D*_3*h*_ form (91.9 kcal/mol) by a commensurate amount.

Since antimony(V) derivatives are significantly more Lewis acidic than their lighter analogues,^7,8^ we have recently started to revisit the chemistry of stiboranes and have paid special attention to structures that are resilient and simple to handle. Such attributes apply to triarylantimonydichlorides, a class of compounds that are typically rather inert, including under ambient conditions. Interestingly, a few reports have suggested that such species may form bimolecular adducts with Lewis bases.^9^ Encouraged by these precedents, we have decided to study the properties of such compounds while also exploring the possibility of Lewis acid enhancement via the imposition of geometrical constraints. In this paper, we compare the properties of Ph_3_SbCl_2_ with those of 5-phenyl-5,5-dichloro-λ^5^-dibenzostibole (**1**),^9a^ which can be regarded as a dichloride analogue of **C** and **D**,^9^ two geometrically constrained compounds known to behave as antimony-based Lewis acids or, synonymously, pnictogen bond donors (Chart 1).^10^

## Results and Discussion

**Figure 1:**
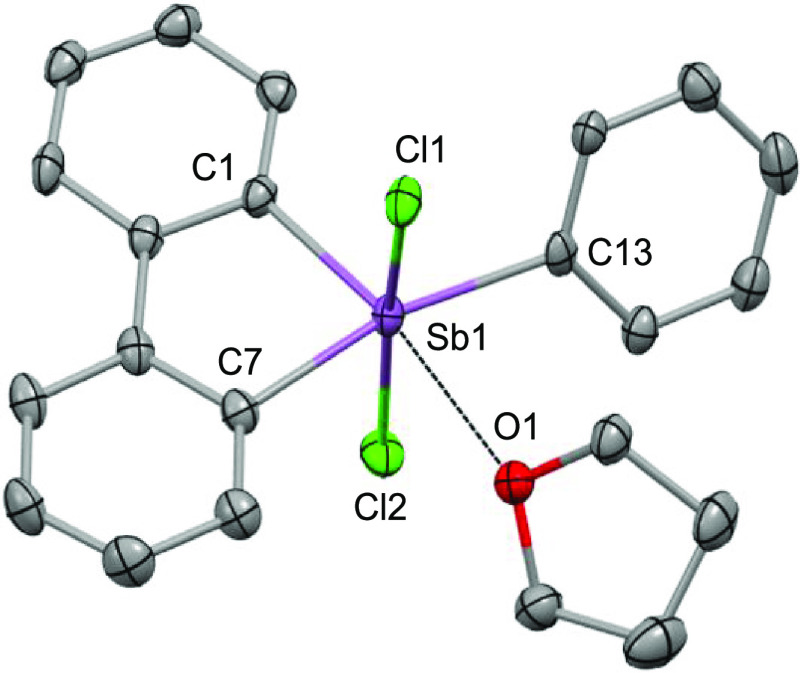
Solid-state representation of **1**-THF as an ORTEP. Hydrogen atoms are omitted for clarity.

**Scheme 1:**
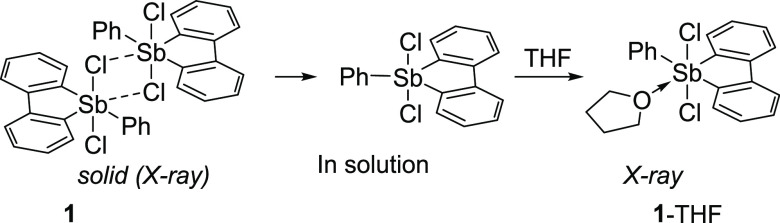
Solid-state and Solution Structure of **1** and Its THF Adduct.

While Ph_3_SbCl_2_ is a monomeric compound, compound **1** was previously shown to exist as a chloride-bridged dimer, as shown in Scheme 1, a feature that already reflects the ground-state destabilization of the structure and the enhanced Lewis acidity of the pnictogen.^9a^ Because of its dimeric nature, **1** is poorly soluble in organic solvents of low polarity, somewhat complicating an evaluation of its Lewis acidic properties. For this reason, we searched for a solvent that could promote dissociation of the dimer and found that addition of tetrahydrofuran (THF) to a solution of **1** in CH_2_Cl_2_ greatly increased the solubility of the compound, suggesting the formation of a THF adduct. Single-crystal X-ray diffraction confirmed the formation of **1**-THF, which features a coordinated THF molecule bound to the antimony center via an Sb–O bond of 2.595(3) Å (Figure 1). The length of this O→Sb dative bond, or pnictogen bond, is comparable to the value of 2.512(4) found in the water adduct of Ph_3_SbO_2_C_6_H_4_.^11^ It is however longer than that in the DMSO adduct of ((*p*-Tol)_3_SbO_2_C_6_H_4_)_2_,^12^ in line with the lower Lewis basicity of THF when compared to that of DMSO. Owing to the presence of this additional THF ligand, the antimony atom of **1**-THF adopts an octahedral geometry, with the two chloride ligands positioned trans from one another and forming a Cl–Sb–Cl angle of 172.34(4)°, close to the ideal value of 180°. Keeping in mind that Ph_3_SbCl_2_ adopts a regular trigonal bipyramidal structure, the obtuse intracyclic C–Sb–C angle of 84.52(2)°, which is typical of such five-membered cyclic structures,^13^ provides a measure of the geometrical constraint imposed by the ring. It is worth noting that with the two chloride ligands in the trans position, the THF ligand is forced to occupy a position trans from one of the Sb–C bonds involved in the five-membered ring. The same can be said about the terminal phenyl ligand that sits trans from the second intracyclic Sb–C bond. Attempts to isolate a THF adduct of Ph_3_SbCl_2_ failed, further illustrating the unique Lewis acidity or pnictogen bond donor properties of **1**.

**Scheme 2:**
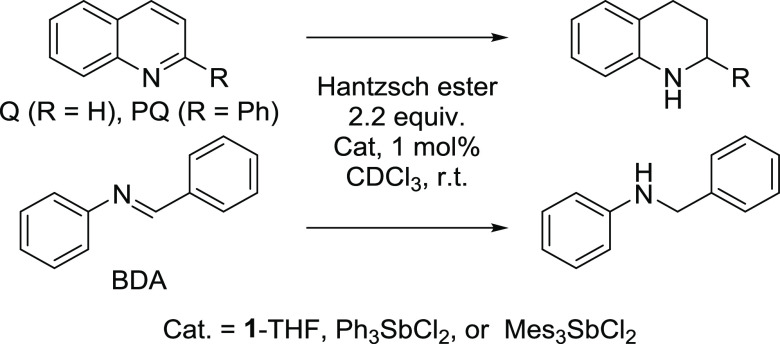
Transfer Hydrogenation Reactions Investigated

In solution, the spectrum of **1**-THF in CH_2_Cl_2_ corresponds to that of a C_2v_ species, suggesting either decoordination of the THF molecule or fast equilibration of the structure. To investigate these possibilities further, we carried out a diffusion-ordered spectroscopy (DOSY) NMR experiment in CDCl_3_, which indicates that THF and **1** diffuse at different rates, with the smaller THF molecule diffusing significantly faster than **1**. To assess the nuclearity of **1** in solution, we also carried out a DOSY experiment in CDCl_3_, which included Ph_3_CH as an internal diffusion standard. This standard was selected because its molecular volume (*V*_mol_ = 1375.7 Å^3^) and solvodynamic radius (*r*_S_ = (3 × *V*_mol_/4 × π)^1/3^ = 6.90 Å) are similar to those of **1** (*V*_mol_ = 2003.2 Å;^3^*r*_S_ = = 7.82 Å). These experiments revealed that Ph_3_CH diffuses slightly faster than **1** (*D*_**1**_/*D*_Ph_3_CH_ = 0.828). This ratio is close to that of the solvodynamic radii (*r*_S(Ph_3_CH)_/*r*_S(**1**)_ = 0.88), which, as per the Stokes–Einstein equation, suggests that **1** indeed exists as a monomer in solution.^14^

**Figure 2:**
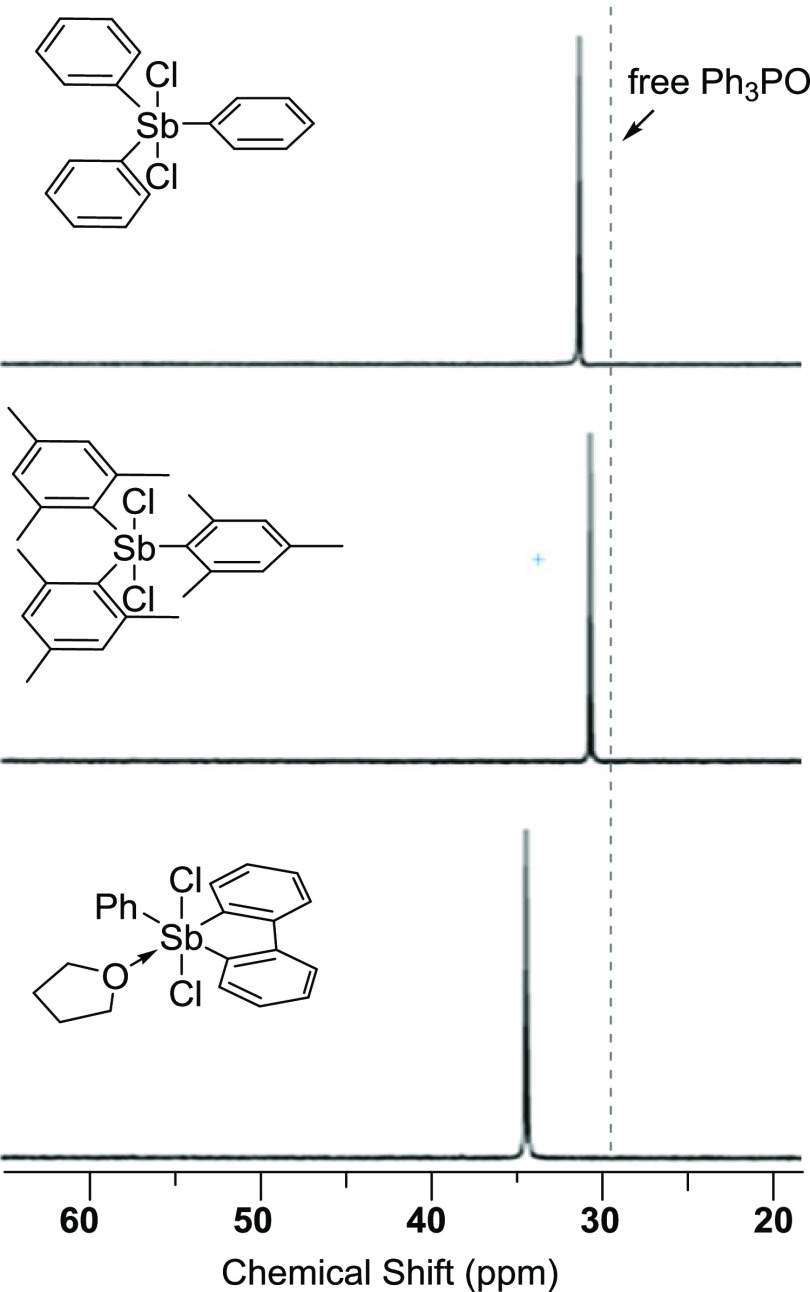
^31^P NMR of Ph_3_SbCl_2_, Mes_3_SbCl_2_, and **1**-THF when treated with 1 equiv of Ph_3_PO in CDCl_3_.

To further investigate the Lewis acidity of **1**, we decided to study its reaction with Ph_3_PO, a Lewis base that we have used previously to probe the Lewis acidity of antimony compounds.^15^ Addition of 1 equiv of **1**-THF to a solution of Ph_3_PO in CDCl_3_ leads to a ^31^P NMR resonance at 34.3 ppm, which is shifted downfield by Δδ = 5.9 ppm when compared to the chemical shift of free Ph_3_PO (29.4 ppm) (Figure 2). Repeating this experiment with Ph_3_SbCl_2_ and Mes_3_SbCl_2_ led to Δδ values of only 1.2 and 0.5 ppm, respectively, reflecting the lower Lewis acidity of these geometrically unconstrained compounds. It is interesting to also note the influence of the bulky mesityl substituents, which appear to almost quench the Lewis acidity of the antimony center of Mes_3_SbCl_2_, as indicated by the smaller Δδ value observed with this Lewis acid.

**Figure 3:**
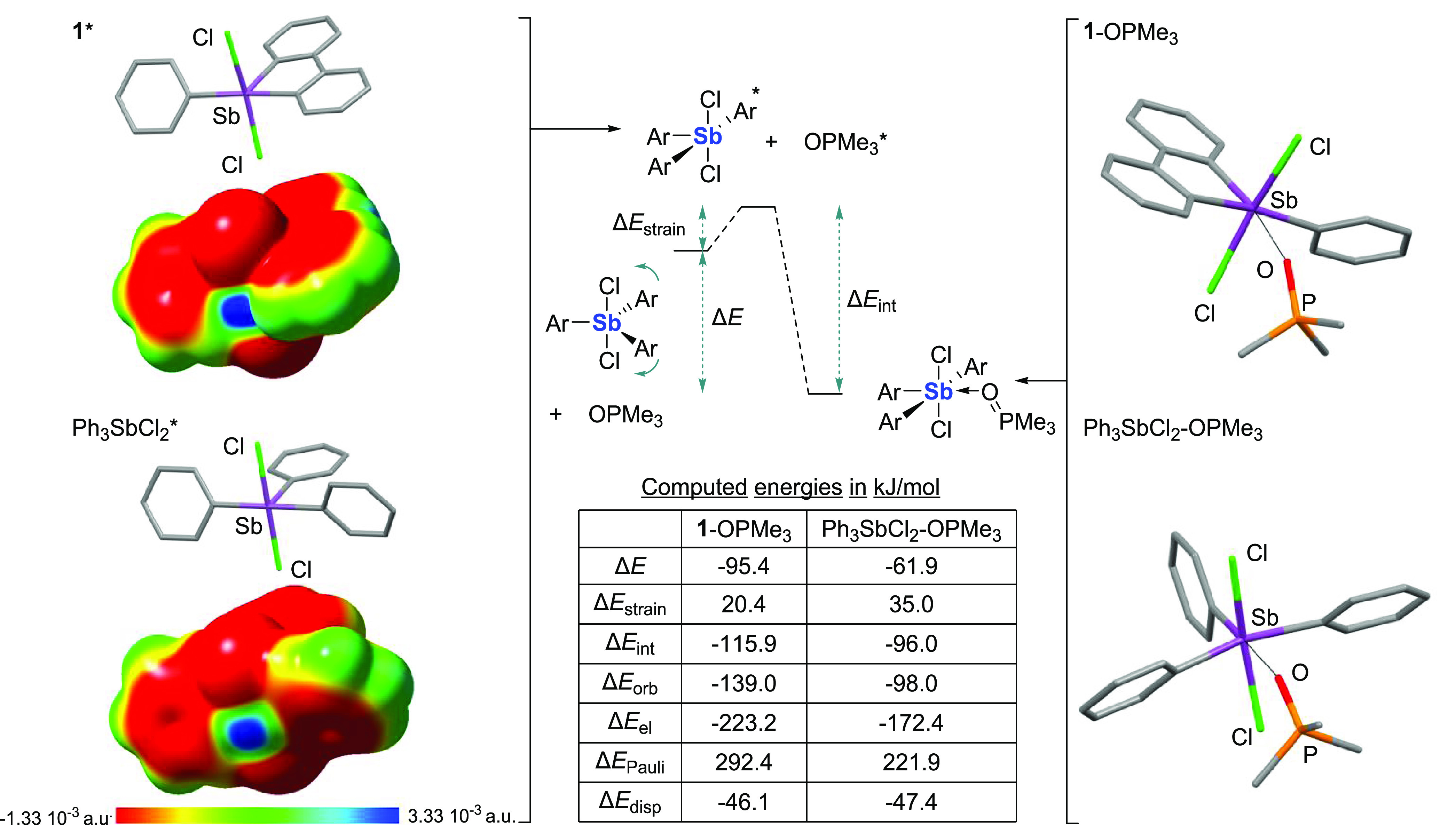
Left: Calculated structure and ESP maps of **1** and Ph_3_SbCl_2_ at the geometry found in their corresponding POMe_3_ adducts. Middle: Diagram illustrating the activation strain and energy decomposition analyses carried out to investigate the energy associated with formation of the POMe_3_ adducts. Right: Optimized structures of the adducts **1**-OPMe_3_ and Ph_3_SbCl_2_-OPMe_3_.

Additional insights into the Lewis acidity of **1** were provided by a simplified activation strain analysis at the equilibrium geometry of the putative adducts **1**-OPMe_3_ and Ph_3_SbCl_2_-OPMe_3_, which were chosen as models due to their structural simplicity. As previously explained,^16^ such an analysis decomposes the energy of an adduct into two terms, namely, Δ*E*_strain_, which corresponds to the energy needed to distort the Lewis acid and the Lewis base to geometries that match those in the adduct, and Δ*E*_int_, the interaction energy of the deformed Lewis-opposite partners (Figure 3). This analysis reveals several noteworthy features. First, the strain energy associated with the deformation of the antimony Lewis acid is significantly lower and thus more favorable in the case of **1** (Δ*E*_strain_ = 20.4 vs 35.0 kJ/mol in the case of Ph_3_SbCl_2_). This result illustrates the benefits that result from the imposition of constraints. As reflected by the obtuse intracyclic C–Sb–C angle that approaches 90°, these constraints force the antimony atom in a coordination geometry closer to that found in the adduct, thereby lowering the energy required to promote **1** to its adduct geometry. A second and possibly more surprising feature is the greater interaction energy Δ*E*_int_ computed in the case of **1** (−116.0 vs −96.0 kJ/mol for Ph_3_SbCl_2_), indicating that the formation of **1**-OPMe_3_ from the deformed components is more favorable by 19 kJ/mol in the case of **1**. To clarify the origin of this difference, both systems were subjected to an energy decomposition analysis^17^ (EDA), which decomposes Δ*E*_int_ into four terms, namely, Δ*E*_orb_, the energy resulting from orbital-based donor–acceptor bonding, Δ*E*_el_, the energy resulting from electrostatic forces, Δ*E*_disp_, the dispersion forces, and Δ*E*_Pauli_, the energy associated with Pauli repulsions. Inspection of the values compiled in the table in Figure 3 indicates that the formation of **1**-OPMe_3_ benefits from significantly larger Δ*E*_orb_ (−139.0 vs −98.0 kJ/mol for Ph_3_SbCl_2_-OPMe_3_) and Δ*E*_el_ terms (−223.2 vs −172.4 kJ/mol for Ph_3_SbCl_2_-OPMe_3_). The Δ*E*_orb_ and Δ*E*_el_ terms correlate with the electrostatic potential surface features of 1* and Ph_3_SbCl_2_*, where the asterisks denote deformed geometry. Indeed, as illustrated in Figure 3, 1* displays a deeper σ hole characterized by a *V*_s,max_ value of 38.4 vs 32.9 kcal/mol for Ph_3_SbCl_2_*. The lower *V*_s,max_ value of Ph_3_SbCl_2_* could be correlated to the two phenyl groups flanking the σ hole since their orientation may allow for greater π donation to the antimony center than in 1*. Last, it is interesting to note that the Pauli repulsion term Δ*E*_Pauli_ is larger and thus less favorable in the case of **1**-OPMe_3_ (292.4 vs 221.9 kJ/mol for Ph_3_SbCl_2_-OPMe_3_). The larger Pauli repulsion term in the case of **1** is readily correlated to the steric shielding of the σ hole by one of the adjacent phenylene units of the biphenyl backbone (Figure 3). However, these effects do not overcome the favorable influence of the orbital and electrostatic terms in the case of **1**-OPMe_3_. The picture that emerges from this activation strain analysis is one in which **1** benefits from both a smaller Δ*E*_strain_, because of its degree of preorganization, and a more negative interaction Δ*E*_int_, the origin of which lies in beneficial orbital and electrostatic energy terms, leading to a greater stabilization of the Me_3_PO adduct (Δ*E* = −95.4 kJ/mol for **1**-OPMe_3_ vs −61.9 kJ/mol for Ph_3_SbCl_2_-OPMe_3_). The stabilizing influence of the orbital terms over the stability of these model complexes serves as a reminder that pnictogen bonds, especially in the case of antimony, benefit from significant Lewis base-to-Lewis acid charge transfer and should not be solely described on the basis of Coulombic forces. The relevance of these charge transfer, orbital-based, and thus covalent interactions is not clearly spelled out in a recently published definition of the pnictogen bond,^18^ despite prior work that showed their unmistakable importance in the case of Pn(III) halides.^10^ Finally, the greater Lewis acidity of **1** is also reflected by its computed fluoride-ion affinity (303.7 kJ/mol), which significantly exceeds that of Ph_3_SbCl_2_ (257.2 kJ/mol) and which approaches that of Ph_3_Sb(O_2_C_6_Cl_4_) (323.1 kJ/mol), another geometrically constrained stiborane.^19^

The elevated Lewis acidity displayed by **1** led us to speculate that this molecule may exhibit enhanced catalytic properties. Inspired by recent advances in pnictogen bond catalysis using both trivalent and pentavalent antimony Lewis acids,^20^ we decided to investigate the use of **1** as a transfer hydrogenation catalyst, using 2-phenyl-quinoline (PQ), quinoline (Q), and *N*-benzylideneaniline (BDA) as substrates and Hantzsch ester as a hydrogen source (Scheme 2). For comparative purposes, we also included Ph_3_SbCl_2_ as a geometrically unconstrained analogue of **1**-THF as well as Mes_3_SbCl_2_ to assess the impact of steric crowding. Reactions were carried out in CDCl_3_, with a 1% catalyst loading and 2.2 equiv of Hantzsch ester. The results of these experiments, which are presented in Table 1, show that **1**-THF is by far the most active in the reduction of PQ, affording 65% conversion after 6 h, a value that greatly exceeds that obtained with Ph_3_SbCl_2_ (15% conversion) at the same time point. The more sterically hindered derivative Mes_3_SbCl_2_ shows essentially no activity, confirming the detrimental effect of steric hindrance in such systems. The results obtained for the reduction of Q mirror those obtained in the case of PQ, with **1**-THF acting as a potent catalyst, while Ph_3_SbCl_2_ and Mes_3_SbCl_2_ show essentially no activity. The reaction involving BDA was more difficult to monitor because of its elevated kinetics. Yet, at the 10 min time point, this reaction was found to be complete with **1**-THF, while those ran with Ph_3_SbCl_2_ and Mes_3_SbCl_2_ showed conversions of 89 and 84%, respectively. The uncatalyzed reaction under the same condition and at the same time point had only progressed to 34% conversion, confirming the role played by the antimony catalysts.

**Table 1:** Compilation of the Results Obtained for the Transfer Hydrogenation of Unsaturated Substrates Using Hantzsch Ester

entry	substrate	Cat.	time	conversion
1	PQ	Ph_3_SbCl_2_	6 h	15%
2	PQ	Mes_3_SbCl_2_	6 h	trace
3	PQ	**1**-THF	6 h	65%
4	Q	Ph_3_SbCl_2_	5 h	trace
5	Q	Mes_3_SbCl_2_	5 h	trace
6	Q	**1**-THF	5 h	62%
7	BDA	Ph_3_SbCl_2_	10 min	89%
8	BDA	Mes_3_SbCl_2_	10 min	84%
9	BDA	**1**-THF	10 min	quantitative

## Conclusions

The results obtained in this study show that triarylantimonydichlorides are latent Lewis acids, which can be enticed to behave as such through the imposition of geometrical constraints. This possibility is illustrated with **1**, which readily forms adducts with Lewis bases while also behaving as a catalyst for the transfer hydrogenation of quinolines and imines. A computational activation strain analysis correlates the Lewis acidity of **1** to the small energy difference between its ground-state structure and that adopted in its Lewis adducts. A strong correlation is also seen with the electrostatic component of the antimony–Lewis base interactions as well as with the charge transfer component of that interaction. The stability and ease of access of triarylantimonydichlorides add to the significance of these findings.

## Experimental Section

### General Information

Ph_3_SbCl_2_ and Mes_3_SbCl_2_ were prepared according to reported procedures.^21^ Solvents were dried by reflux under N_2_ over Na/K (pentane and THF). All other solvents were used as received. Commercially available chemicals were purchased and used as provided (commercial sources: Aldrich for SbCl_3_, Matrix Scientific for biphenyl, and TCI Chemicals for Ph_3_PO). Ambient-temperature NMR spectra were recorded on a Varian Unity Inova 500 FT, a Bruker Avance 500 NMR spectrometer, or a Varian VnmrS 500 for the DOSY experiments (500 MHz for ^1^H and 126 MHz for ^13^C). A Bruker Ascend 400 NMR spectrometer (400 MHz for ^1^H and 101 MHz for ^13^C) was also used for some of the spectra. ^1^H and ^13^C NMR chemical shifts are given in ppm and are referenced against SiMe_4_ using residual solvent signals used as secondary standards. Elemental analyses were performed at Atlantic Microlab (Norcross, GA).

### Computational Details

Density functional theory (DFT) structural optimizations were performed using the Gaussian 16 program.^22^ The optimizations were carried out using the B3LYP functional and the following mixed basis set: Sb cc–pVTZ–PP; P/O/Cl: 6–31g(d’); H/C: 6–31g. Optimized structures had their structures and molecular orbitals rendered using the Avogadro program.^23^ Frequency calculations were used to confirm that optimization had converged to true minima. The optimized structures (available as xyz files submitted as the Supporting Information, SI) are in excellent agreement with the solid-state structures. All thermochemical analyses, including EDAs, were carried out using the ADF software with B3LYP-D3 as the functional and the QZ4P as the basis set.^24^ Energy values were calculated for the separate molecules using single-point calculations within the software using the same basis sets and level of theory. Fragmentation was completed using trimethyl phosphine oxide and the given stiborane within the software, using the energy decomposition analysis subroutine. The Avogadro program^23^ was used for visualization of the optimized geometries. The enthalpies used to derive the FIA were obtained by single-point calculations carried out at the optimized geometry with the B3LYP functional and the following mixed basis sets: aug-cc-pVTZ-pp for Sb and 6-311+g(2d,p) for C, H, and F. The enthalpy correction term was obtained from the above-mentioned frequency calculations.

### Crystallographic Measurements

The crystallographic measurements for **1**-THF were performed at 110(2) K using a Bruker D8 QUEST diffractometer (Mo Kα radiation, λ = 0.71069 Å) equipped with a Photon III detector. A specimen of suitable size and quality was selected and mounted onto a nylon loop. The structure was solved by direct methods, which successfully located most of the non-hydrogen atoms. Semiempirical absorption corrections were applied. Subsequent refinement on F^2^ using the SHELXTL/PC package (version 6.1) allowed location of the remaining non-hydrogen atoms.

### Synthesis of **1**-THF

A CH_2_Cl_2_ solution (20 mL) of 5-phenyl-λ^3^-dibenzostibole^9a^ (450.0 mg, 1.066 mmol) was treated with 1 equiv of phenyl iodine dichloride (355.0 mg, 1.291 mmol) dissolved in 10 mL of CH_2_Cl_2_. The resulting mixture was stirred for 2 h, and then, the organic solvent was evaporated. The resulting oil was dissolved in 5 mL of CH_2_Cl_2_ and recrystallized by the addition of 10 mL of n-pentane. The resulting powder was then washed with n-pentane 3 × 5 mL. The powder was then dissolved in 5 mL of THF and heated to 65 °C. To this solution, pentane was added dropwise (5 mL) until a precipitate formed. The precipitate was dried under vacuum, and the resulting compound was isolated as **1**-THF (404 mg, 0.817 mmol, 76.7% yield). Single crystals of **1**-THF suitable for X-ray diffraction were grown by vapor diffusion of *n*-pentane (5 mL) into a concentrated solution of **1**-THF (40 mg, 0.081 mmol) in THF (1 mL). ^1^H NMR (499.42 MHz; CD_2_Cl_2_): δ 8.3 (m, 2H *o*-phenyl), 8.21 (d, *J*_H–H_ = 7.4 Hz, 2H), 7.95 (d, *J*_H–H_ = 7.4 Hz, 2H), 7.67–7.59 (m, 5H), 7.54 (t, *J*_H–H_ = 7.3 Hz, 2H), 3.7 (m, 4H), 1.82 (m, 4H), ^13^C{^1^H} NMR (125.58 MHz; CD_2_Cl_2_): 141.8 (s), 137.8 (s), 133.9 (s), 132.2 (s), 132 (s), 130.8 (s), 130.1 (s), 129.7 (s), 123.8 (s), 131.2 (s), 68.0 (s), 25.4 (s) Elemental analysis was obtained on a recrystallized sample. Calcd (%) for C_18_H_13_Cl_2_Sb-0.35 (C_4_H_8_O): C, 52.11; H, 3.56. Found: C, 51.68; H, 4.18. These results indicate partial loss of the THF ligand during handling and shipping.

### Coordination of Ph_3_PO to Lewis Acids

Separate samples of Ph_3_SbCl_2_ (10 mg, 24 μmol), Mes_3_SbCl_2_ (13 mg, 24 μmol), and freshly prepared **1**-THF (11.5 mg, 23.3 μmol) were dissolved in 1 mL of CH_2_Cl_2_. To these three separate solutions, Ph_3_PO (7.0 mg, 25 μmol) was added, and the resulting solutions were subjected to ^31^P{^1^H}NMR spectroscopy. The spectra shown in Figure 2 each averaged 512 scans.

### Catalysis

In a typical experiment, 2-phenyl-quinoline (208 mg, 1.014 mmol), Hantzsch ester (566 mg, 2.23 mmol), and the catalyst (1 mol %) were combined in CDCl_3_ (2.0 mL) and transferred to a vial. The reaction solution was stirred constantly to ensure sufficient mixing of the heterogeneous solution. The progress of the catalysis was monitored by ^1^H NMR spectroscopy. The conversions were calculated by NMR integration. A similar protocol was followed for the other substrates. The spectra corresponding to the experiments compiled in Table 1 are provided in the SI.

## Supporting Information Available

Additional experimental and computational details (PDF)
Optimized structures in xyz format (XYZ)

## Supplementary Material

om2c00565_si_001.pdf

om2c00565_si_002.xyz
