# Supplementary material for: Are Ar3SbCl2 Species Lewis Acidic? Exploration of the Concept and Pnictogen Bond Catalysis Using a Geometrically Constrained Example
Source: Organometallics. 2023 Jan 30;42(3):240–5. doi: 10.1021/acs.organomet.2c00565 (PMC10848295; doi:10.1021/acs.organomet.2c00565)
Supplement: Supplementary file 1 — om2c00565_si_001.pdf [file om2c00565_si_001.pdf]

Supporting Information

Are Ar_3SbCl_2 species Lewis acidic? Exploration of the concept and pnictogen bond catalysis using a geometrically constrained example

Jesse E. Smith, and François P. Gabbaï*
Department of Chemistry, Texas A&M University, College Station, TX 77843-3255
**Email: francois@tamu.edu*

Table of content

Figure S1. ^{1}H NMR spectrum of Ph_3SbCl_2 in $CDCl_3$ S2
Figure S2. ^{1}H NMR spectrum of Mes_3SbCl_2 in $CDCl_3$ S2
Figure S3. ^{1}H NMR spectrum of **1**-THF in $CDCl_3$ S3
Figure S4. ^{1}H NMR spectrum of the aromatic region in **1**-THF in $CDCl_3$ S3
Figure S5. $^{13}C\{^1H\}$ NMR spectrum of **1**-THF in CD_2Cl_2 with the solvent peak truncated. S4
Figure S6. ^{1}H DOSY experiment for **1**-THF at room temperature measuring the diffusion of **1** and THF. S4
Figure S7. ^{1}H NMR spectrum obtained 6 h into the reaction of 2-phenyl-quinoline and Hantzsch ester with Ph_3SbCl_2 as the catalyst. S5
Figure S8. ^{1}H NMR spectrum obtained 6 h into the reaction of 2-phenyl-quinoline and Hantzsch ester with Mes_3SbCl_2 as the catalyst. S5
Figure S9. ^{1}H NMR spectrum obtained 6 h into the reaction of 2-phenyl-quinoline and Hantzsch ester with **1**-THF as the catalyst. S6
Figure S10. ^{1}H NMR spectrum obtained 5 h into the reaction of quinoline and Hantzsch ester with Ph_3SbCl_2 as a catalyst. S6
Figure S11. ^{1}H NMR spectrum obtained 5 h into the reaction of quinoline and Hantzsch ester with Mes_3SbCl_2 as a catalyst. S7
Figure S12. ^{1}H NMR spectrum obtained 5 h into the reaction of quinoline and Hantzsch ester with **1**-THF as a catalyst. S7
Figure S13. ^{1}H NMR spectrum obtained 10 min into the reaction of N-benzylideneaniline and Hantzsch ester with Ph_3SbCl_2 as a catalyst. S8
Figure S14. ^{1}H NMR spectrum obtained 10 min into the reaction of N-benzylideneaniline and Hantzsch ester with Mes_3SbCl_2 as a catalyst. S8
Figure S15. ^{1}H NMR spectrum obtained 10 min into the reaction N-benzylideneaniline and Hantzsch ester with **1**-THF as a catalyst. S9
Figure S16. ^{1}H NMR spectrum obtained 10 min into the reaction of N-benzylideneaniline and Hantzsch ester without a catalyst. S9
Figure S17. Sample from the output file with the energy decomposition analysis results of **1**-Me_3PO with A. total Pauli repulsion energy; B. electrostatic interaction energy; C. orbital overlap energy; D. dispersion energy and, E. total bonding energy. Results are shown in Figure 3 of the main text. S10

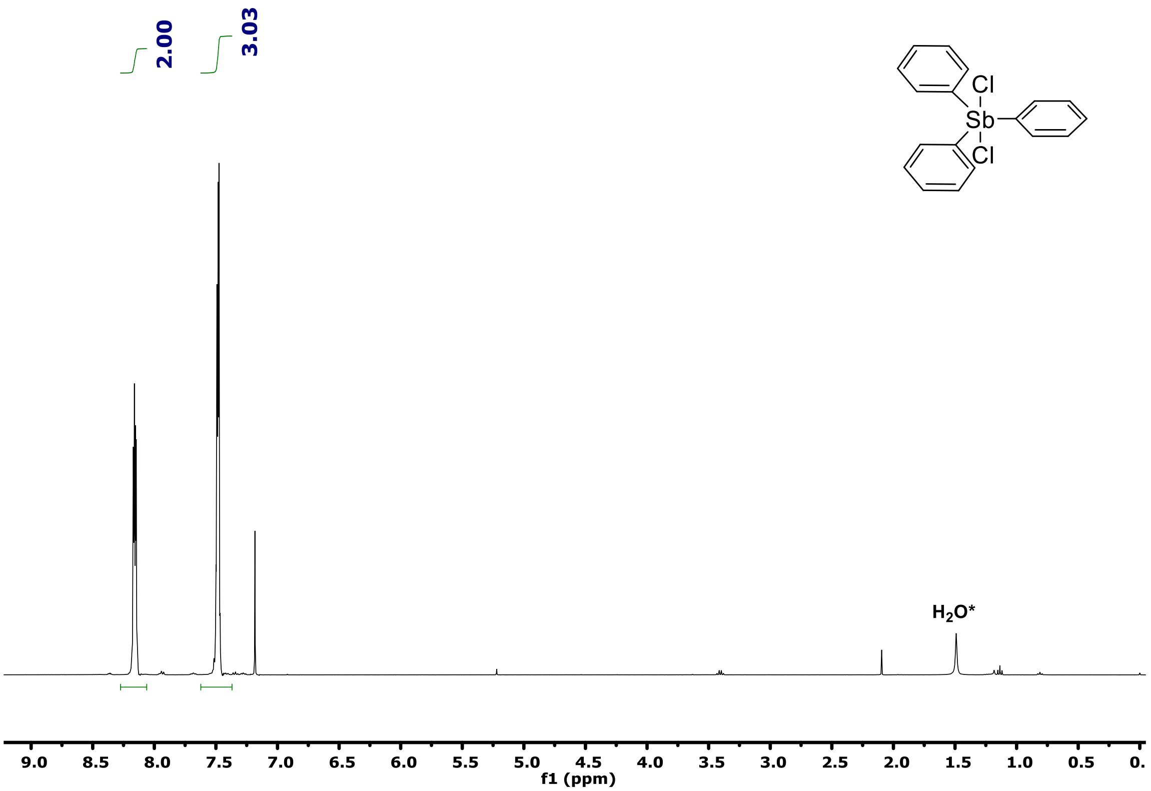

Figure S1. ^{1}H NMR spectrum of Ph_3SbCl_2 in $CDCl_3$

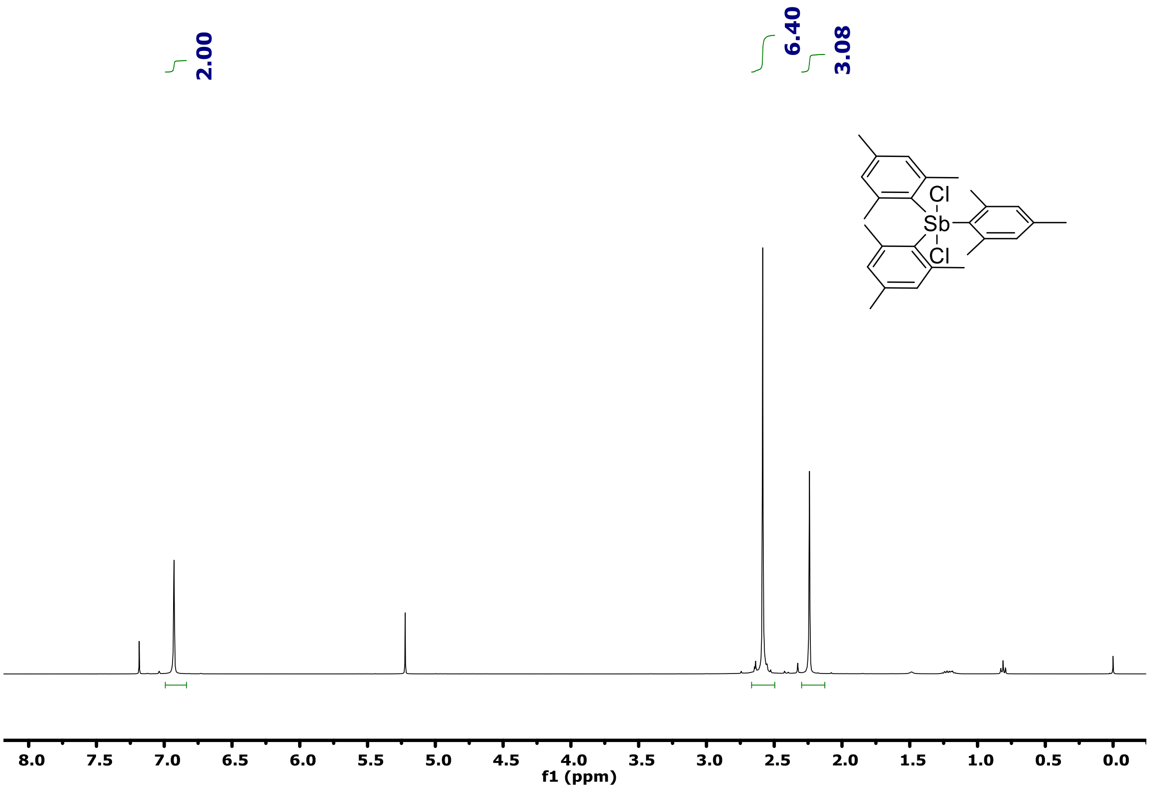

Figure S2. ^{1}H NMR spectrum of Mes_3SbCl_2 in $CDCl_3$

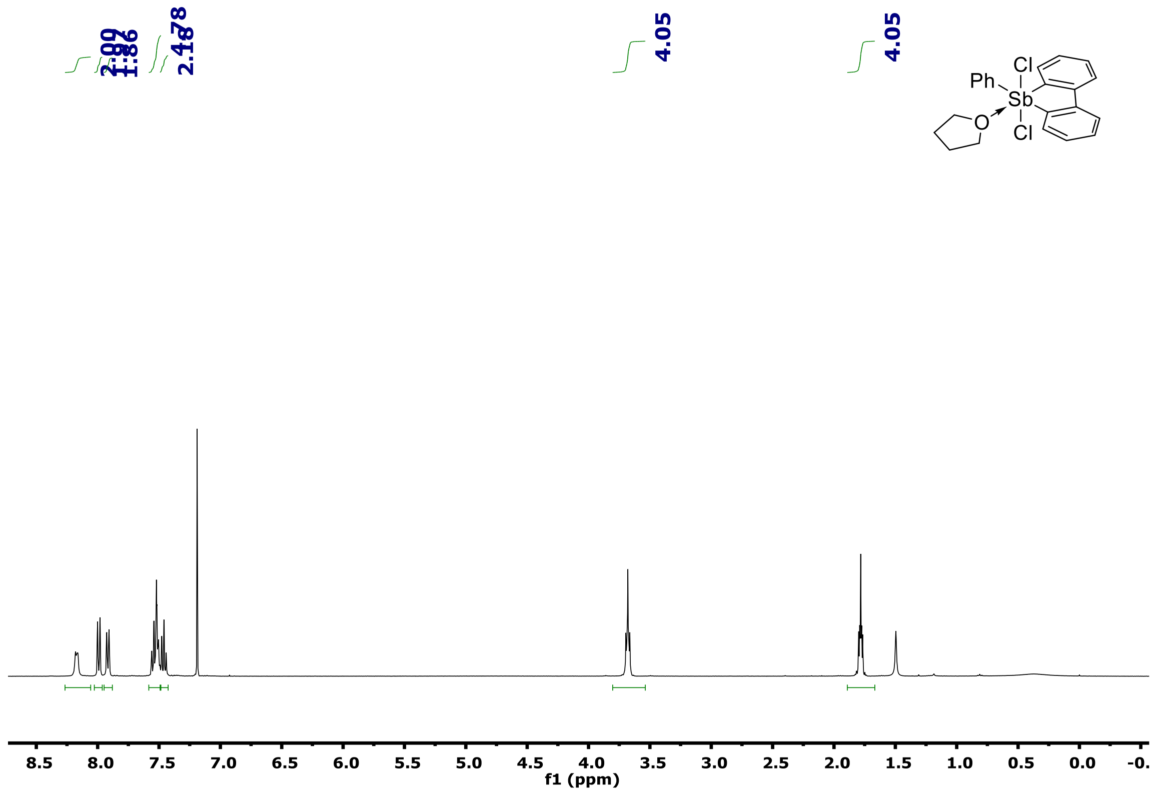

Figure S3. ^{1}H NMR spectrum of **1**-THF in $CDCl_3$

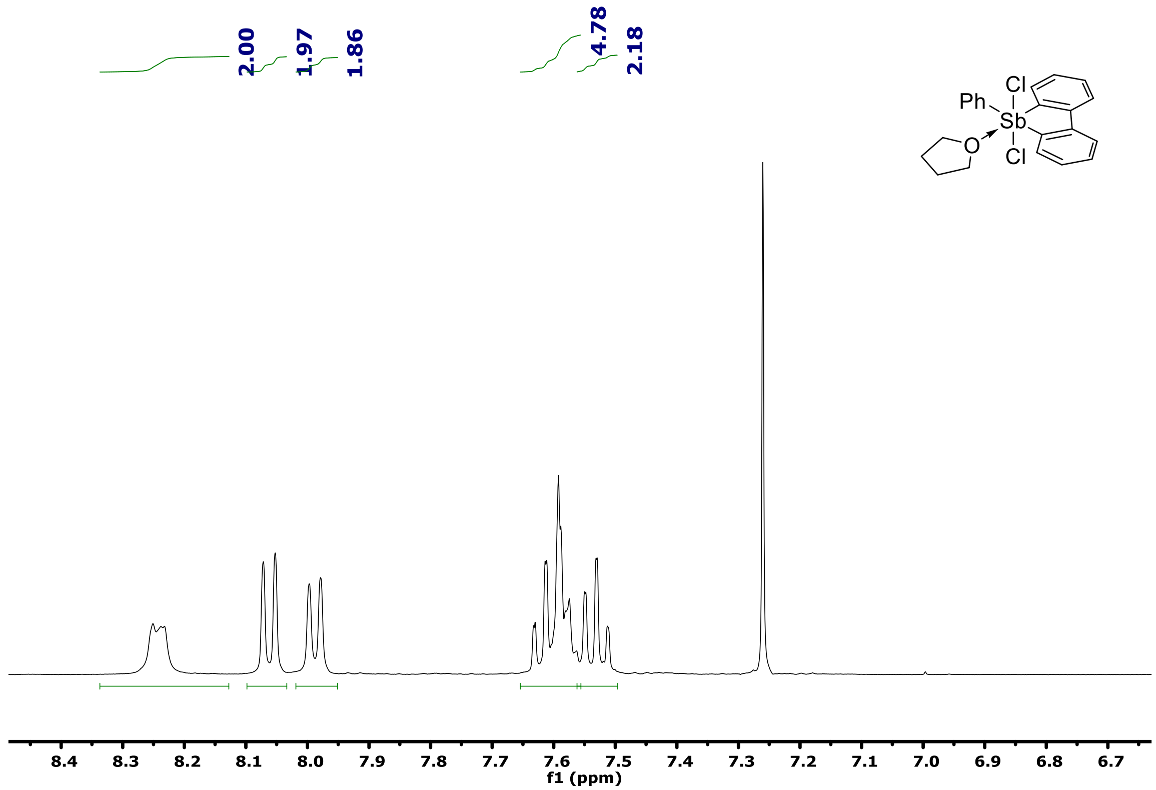

Figure S4. ^{1}H NMR spectrum of the aromatic region in **1**-THF in $CDCl_3$

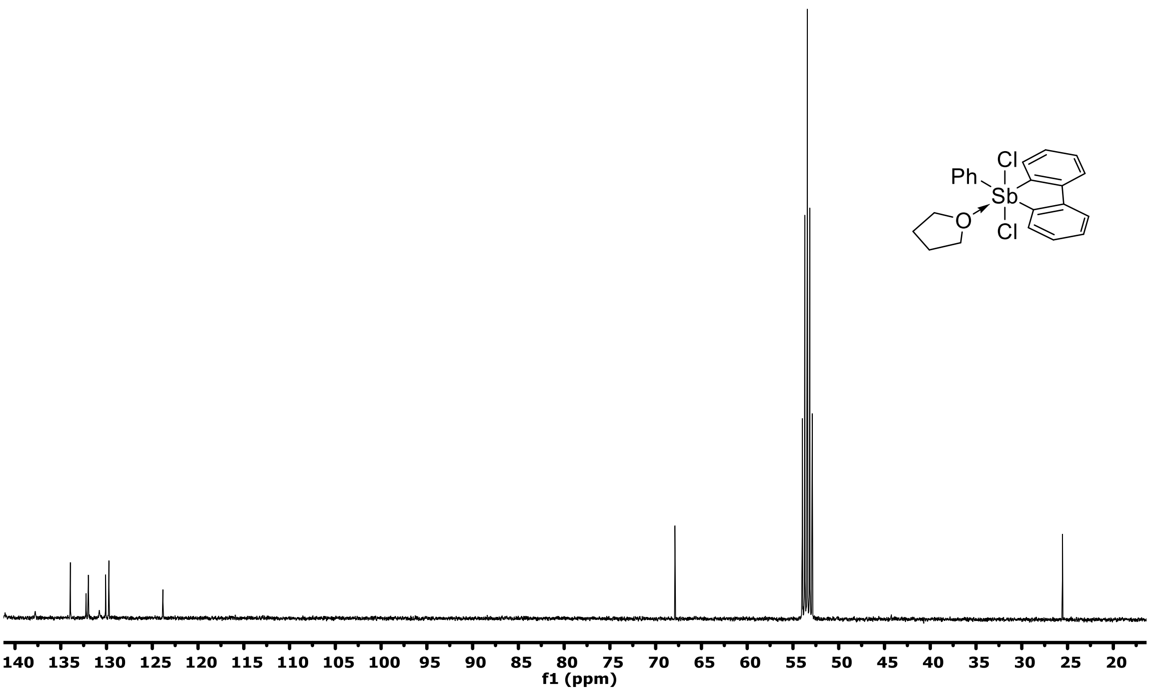

Figure S5. $^{13}C\{^{1}H\}$ NMR spectrum of **1**-THF in CD_2Cl_2 with the solvent peak truncated.

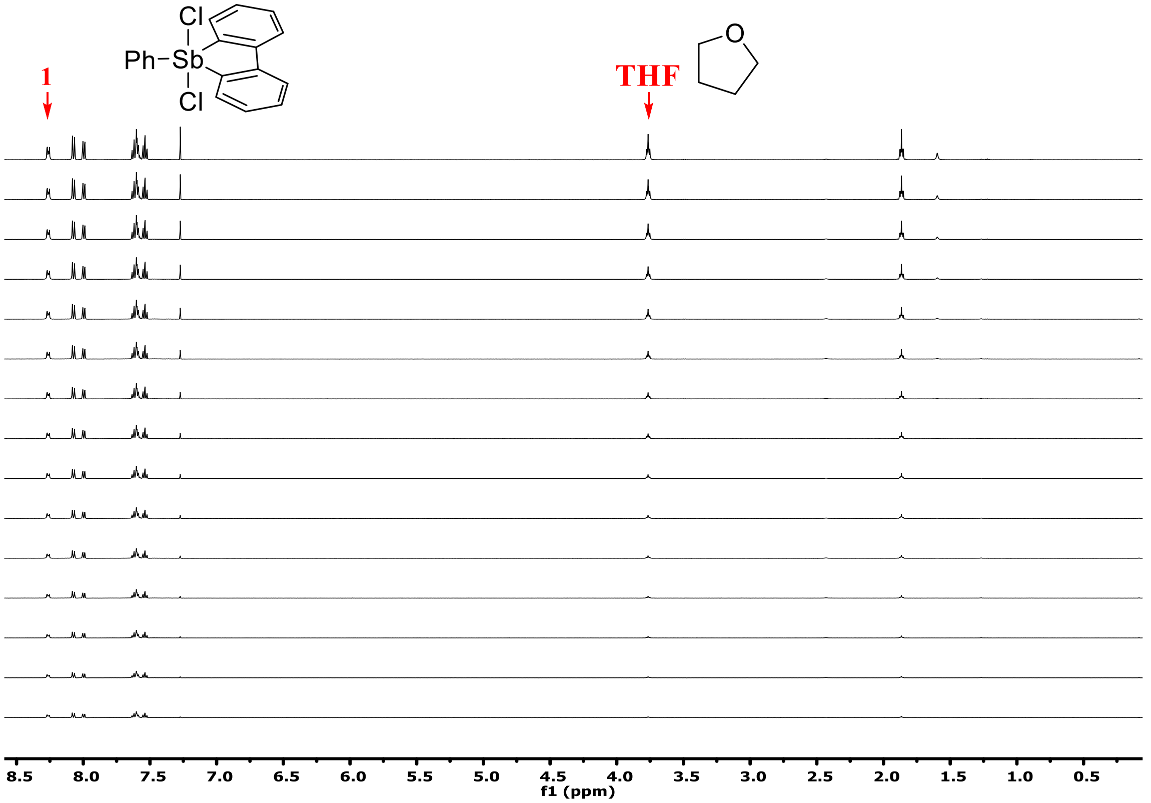

Figure S6. ^{1}H DOSY experiment for **1**-THF at room temperature measuring the diffusion of **1** and THF.

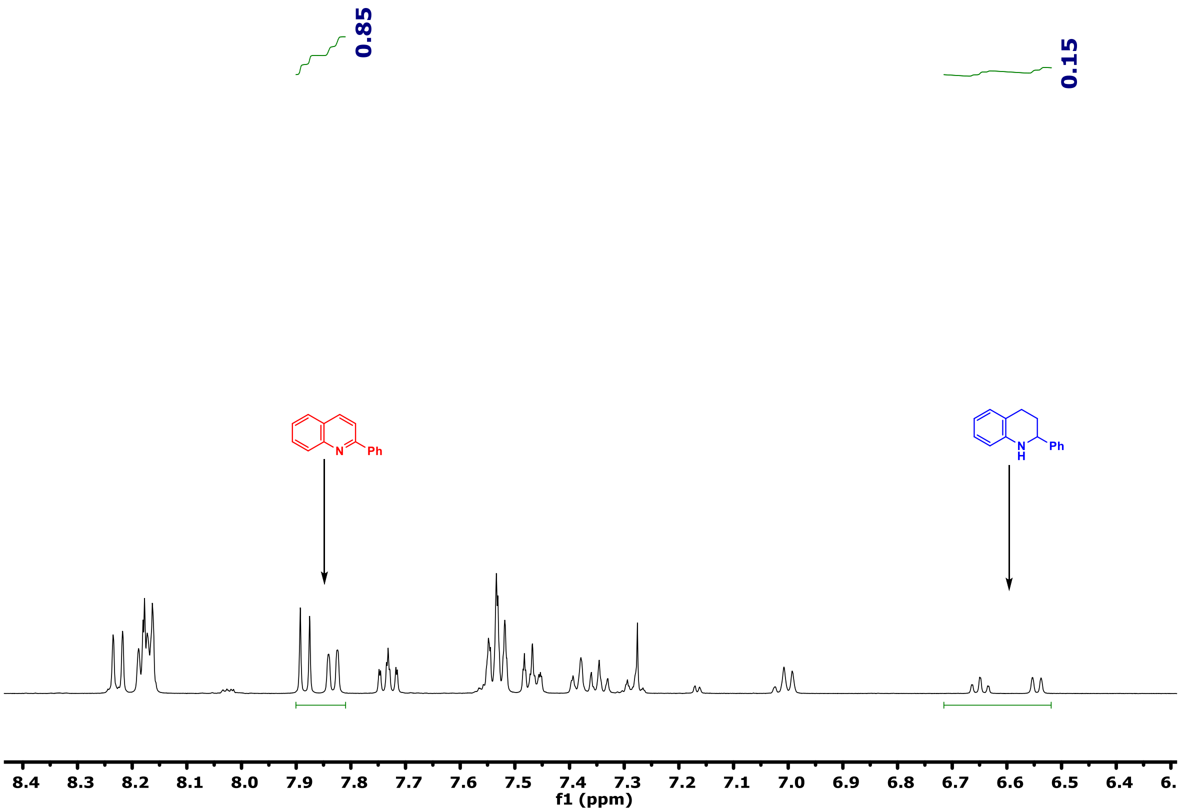

Figure S7. ^{1}H NMR spectrum obtained 6 h into the reaction of 2-phenyl-quinoline and Hantzsch ester with Ph_3SbCl_2 as the catalyst.

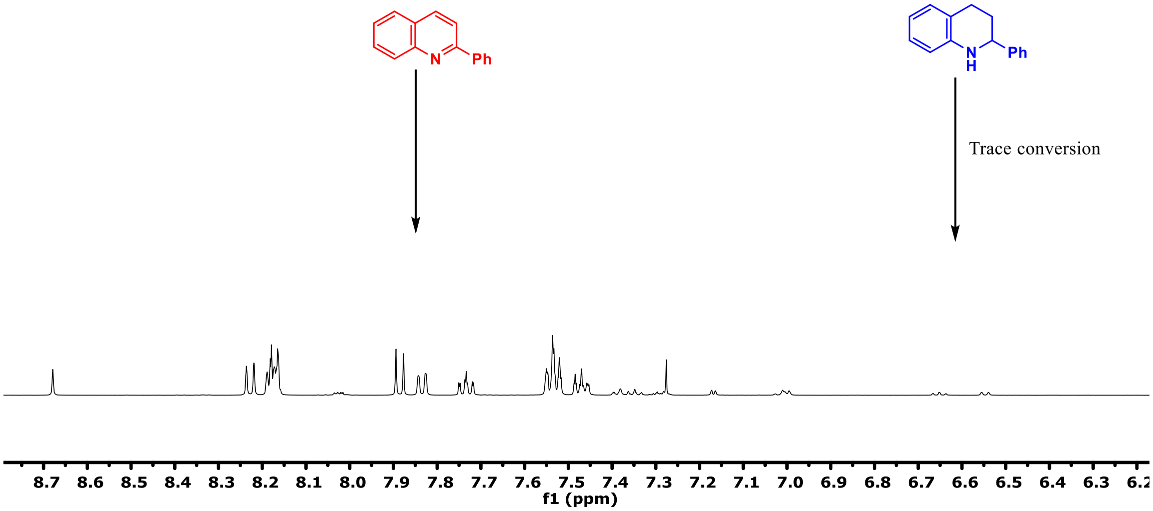

Figure S8. ^{1}H NMR spectrum obtained 6 h into the reaction of 2-phenyl-quinoline and Hantzsch ester with Mes_3SbCl_2 as the catalyst.

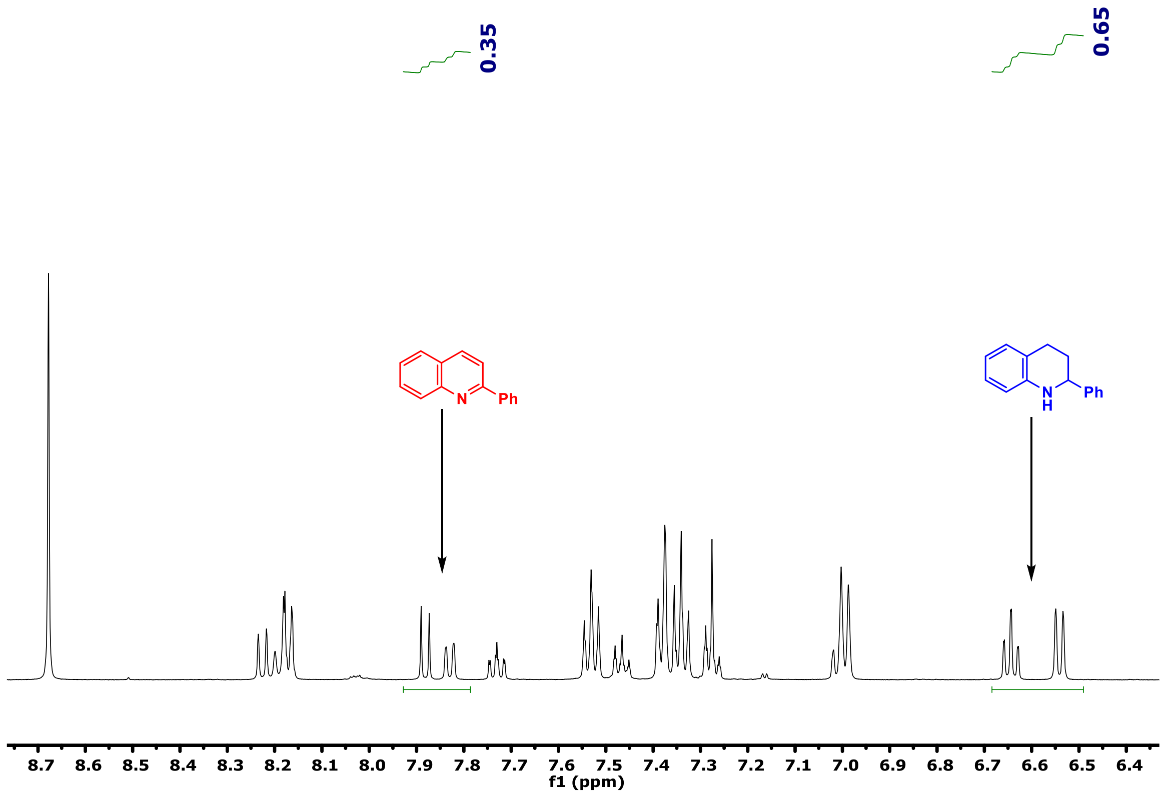

Figure S9. ^{1}H NMR spectrum obtained 6 h into the reaction of 2-phenyl-quinoline and Hantzsch ester with **1**-THF as the catalyst.

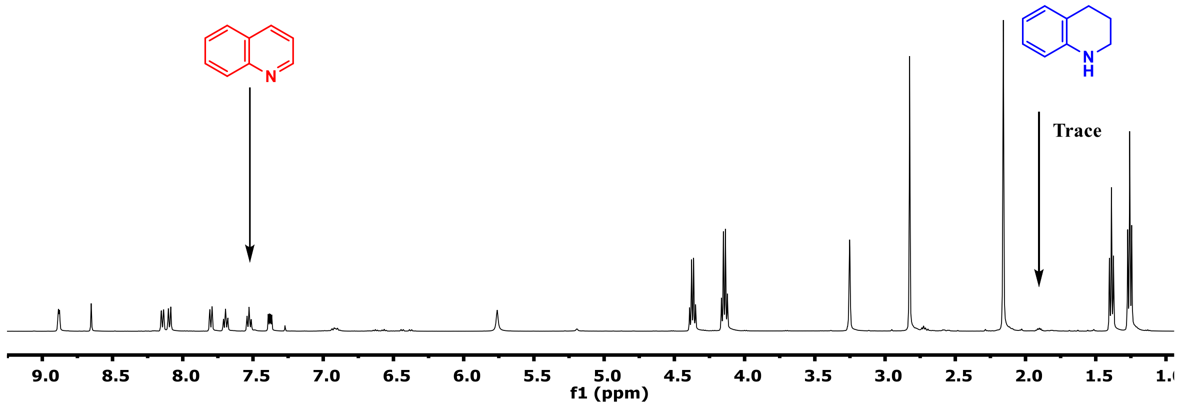

Figure S10. ^{1}H NMR spectrum obtained 5 h into the reaction of quinoline and Hantzsch ester with Ph_3SbCl_2 as a catalyst.

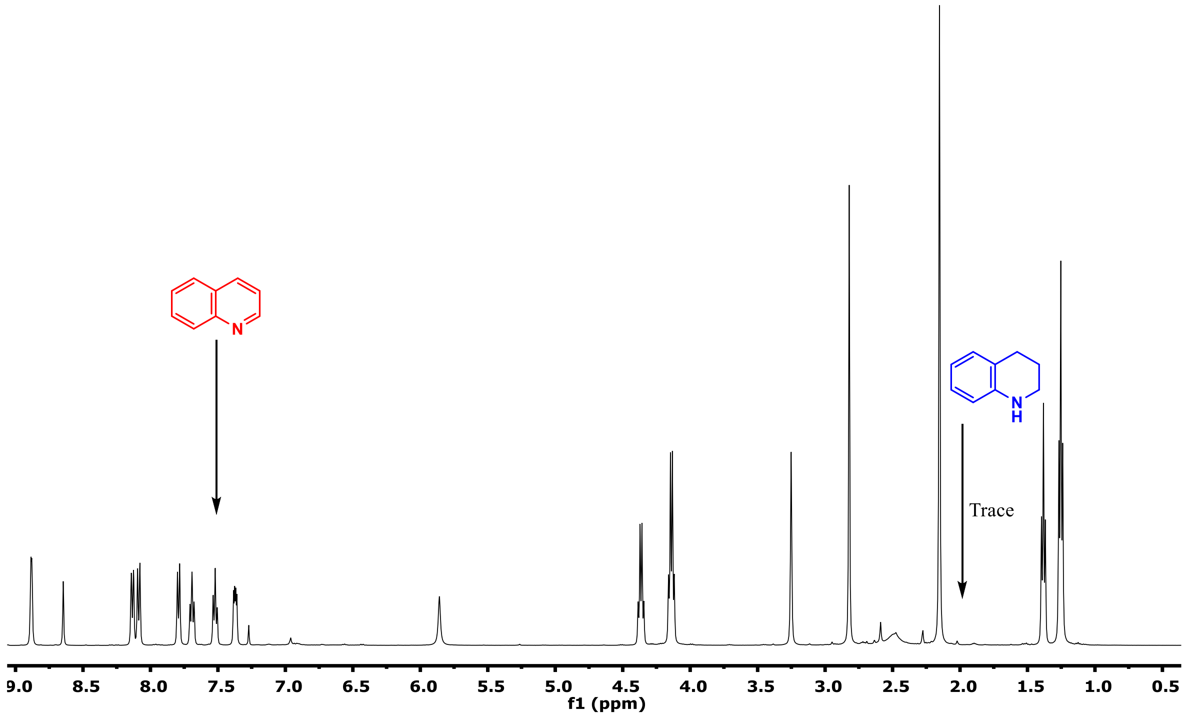

Figure S11. ^{1}H NMR spectrum obtained 5 h into the reaction of quinoline and Hantzsch ester with Mes_3SbCl_2 as a catalyst.

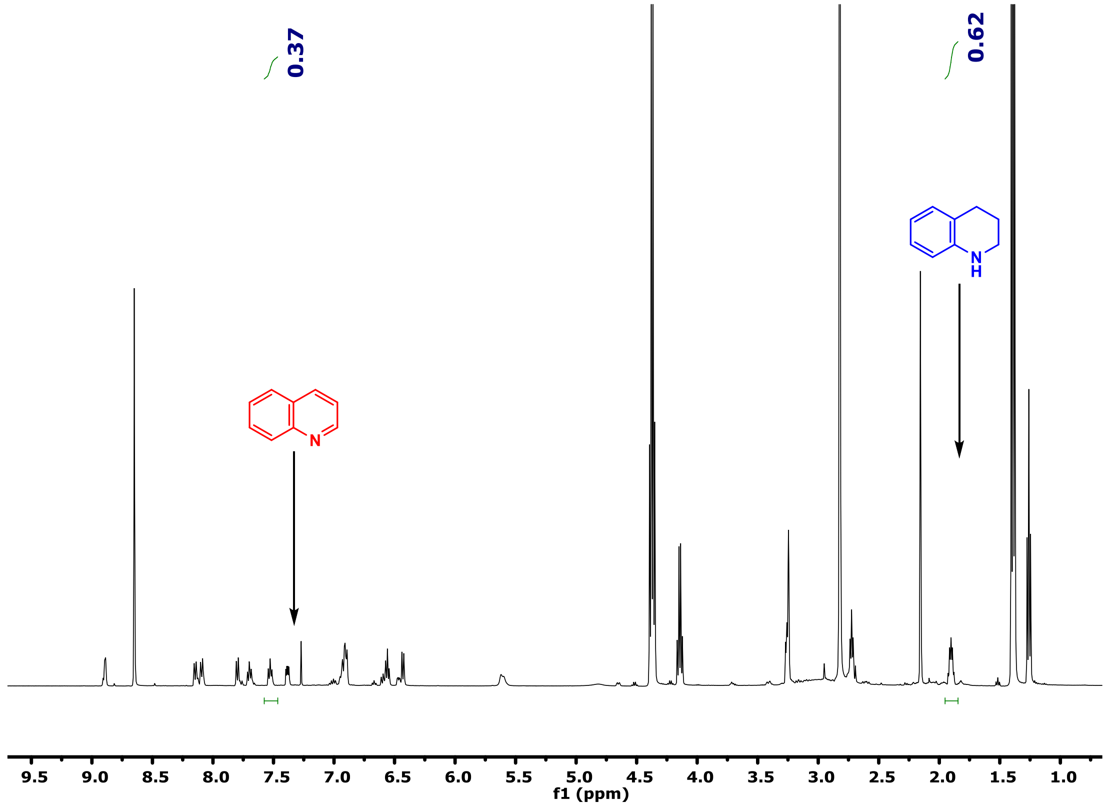

Figure S12. ^{1}H NMR spectrum obtained 5 h into the reaction of quinoline and Hantzsch ester with **1**-THF as a catalyst.

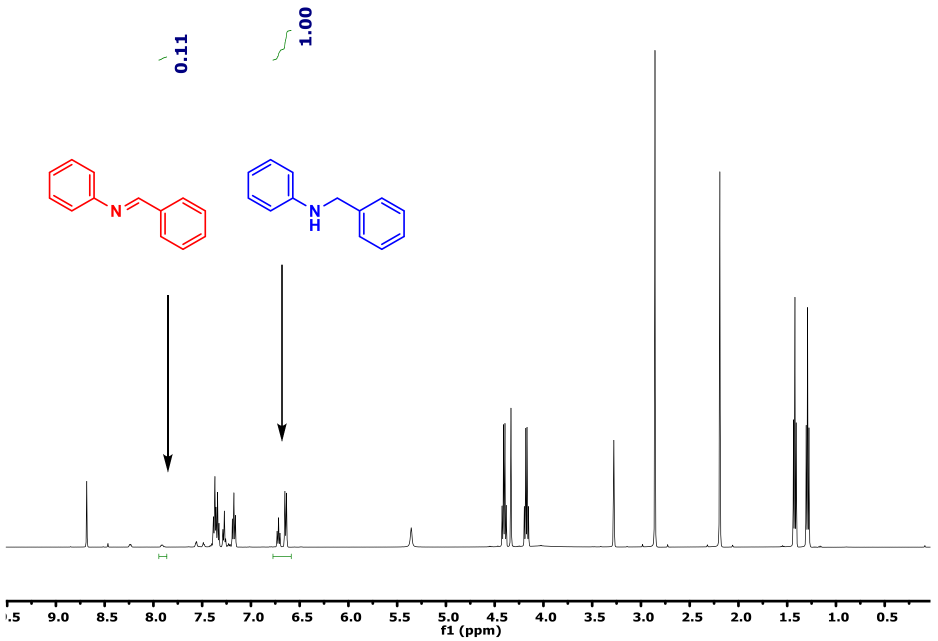

Figure S13. ^{1}H NMR spectrum obtained 10 min into the reaction of N-benzylideneaniline and Hantzsch ester with Ph_3SbCl_2 as a catalyst.

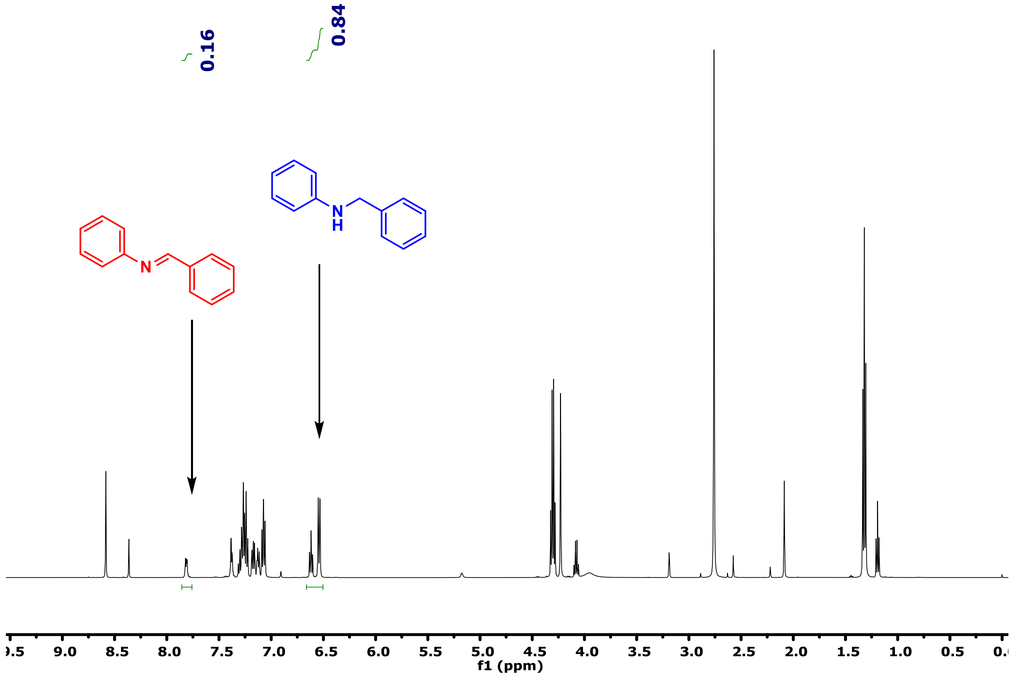

Figure S14. ^{1}H NMR spectrum obtained 10 min into the reaction of N-benzylideneaniline and Hantzsch ester with Mes_3SbCl_2 as a catalyst.

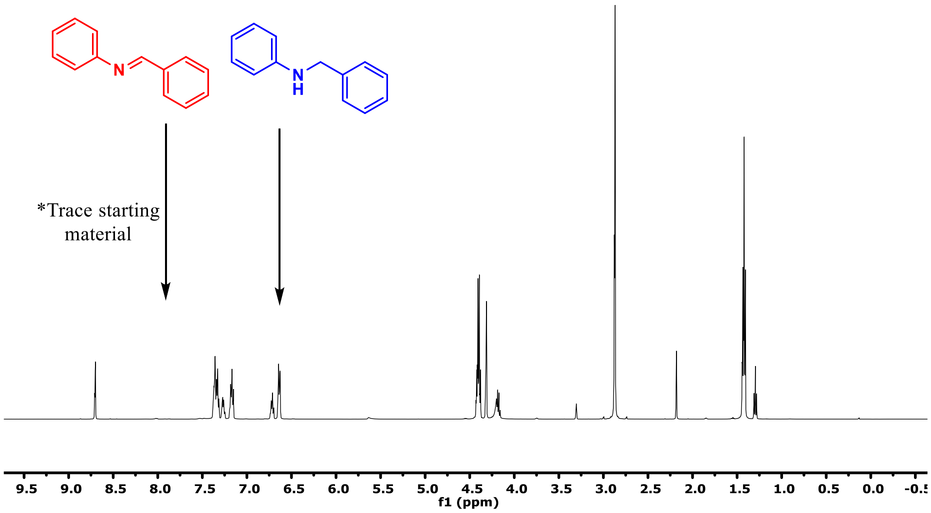

Figure S15. ^{1}H NMR spectrum obtained 10 min into the reaction N-benzylideneaniline and Hantzsch ester with **1**-THF as a catalyst.

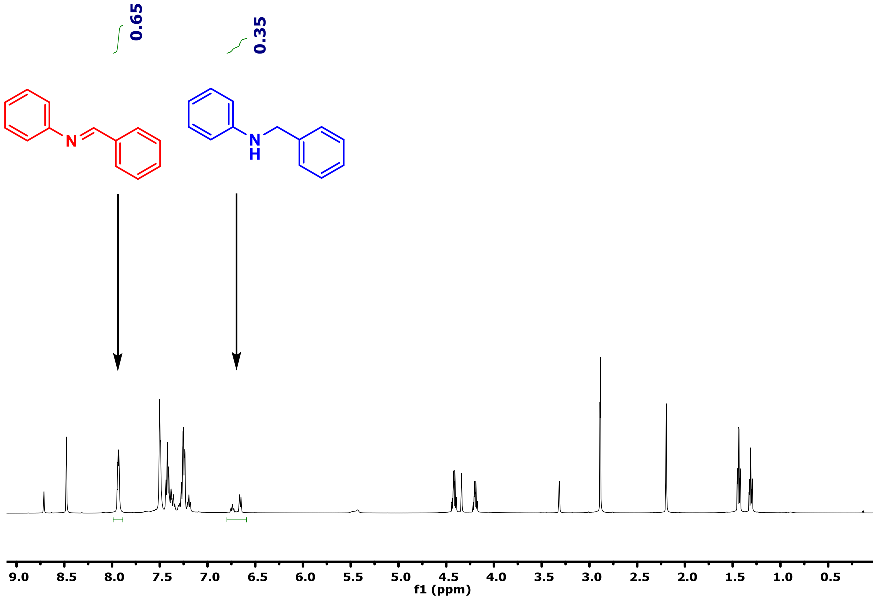

Figure S16. ^{1}H NMR spectrum obtained 10 min into the reaction of N-benzylideneaniline and Hantzsch ester without a catalyst.

```
                                                  hartree            eV     kcal/mol        kJ/mol
                                     --------------------  ------------  -----------  ------------
    pauli hf:                        -0.000107897160689       -0.0029        -0.07         -0.28
    elstat hf:                       -0.028169613435807       -0.7665       -17.68        -73.96

  Pauli Repulsion
    Kinetic (Delta T^0):              0.890141625432794       24.2220       558.57       2337.07
    Delta V^Pauli Coulomb:           -0.629720500147744      -17.1356      -395.16      -1653.33
    Delta V^Pauli Hybrid-X:          -0.100398186276493       -2.7320       -63.00       -263.60
    Delta V^Pauli Hybrid-C:          -0.020381717098198       -0.5546       -12.79        -53.51
    Delta V^Pauli HF-Exchange:       -0.028277510596496       -0.7695       -17.74        -74.24
                                     --------------------  ------------  -----------  ------------
A.  Total Pauli Repulsion:            0.111363711313863        3.0304        69.88        292.39
   (Total Pauli Repulsion =
    Delta E^Pauli in BB paper)

  Steric Interaction
    Pauli Repulsion (Delta E^Pauli):  0.111363711313863        3.0304        69.88        292.39
B.  Electrostatic Interaction:       -0.084995592316318       -2.3128       -53.34       -223.16
   (Electrostatic Interaction =
    Delta V_elstat in the BB paper)
                                     --------------------  ------------  -----------  ------------
    Total Steric Interaction:         0.026368118997545        0.7175        16.55         69.23
   (Total Steric Interaction =
    Delta E^0 in the BB paper)

  Orbital Interactions
    A:                               -0.053039231629630       -1.4433       -33.28       -139.25
    HF orbital correction:            0.000081765143256        0.0022         0.05          0.21
                                     --------------------  ------------  -----------  ------------
C.  Total Orbital Interactions:      -0.052957466486375       -1.4410       -33.23       -139.04

  Alternative Decomposition Orb.Int.
    Kinetic:                         -0.727798529856902      -19.8044      -456.70      -1910.83
    Coulomb:                          0.620033182475755       16.8720       389.08       1627.90
    XC+HF:                            0.054807880894773        1.4914        34.39        143.90
    XC only:                          0.046972813138256        1.2782        29.48        123.33
    HF Orbital:                       0.007835067756517        0.2132         4.92         20.57
                                     --------------------  ------------  -----------  ------------
    Total Orbital Interactions:      -0.052957466486374       -1.4410       -33.23       -139.04

    Residu (E=Steric+OrbInt+Res):     0.000000531215170        0.0000         0.00          0.00
D.  Dispersion Energy:               -0.017570517188631       -0.4781       -11.03        -46.13
E. Total Bonding Energy:             -0.044159333462291       -1.2016       -27.71       -115.94
```

Figure S17. Sample from the output file with the energy decomposition analysis results of **1**-Me_3PO with A. total Pauli repulsion energy; B. electrostatic interaction energy; C. orbital overlap energy; D. dispersion energy and, E. total bonding energy. Results are shown in Figure 3 of the main text.
